# Initial arterial access used and associated factors in coronary procedures: A retrospective study from a tertiary indian center

**DOI:** 10.1371/journal.pone.0344998

**Published:** 2026-03-18

**Authors:** Abhijit Vilas Kulkarni, Adefres Bahiru, D. Manjunath, Mohan J. Murali, Hina Khaleel

**Affiliations:** 1 Interventional Cardiologist & Electrophysiologist, Apollo Hospital, Bangalore, India; 2 Internist Cardiologist, Addis Cardiac Hospital, Addis Ababa, Ethiopia; 3 Consultant Cardiologist, Apollo Hospital, Bangalore, India; 4 Apollo Hospital, Bangalore, India; 5 Apollo Hospital, Bangalore, India; Ataturk University Faculty of Medicine, TÜRKIYE

## Abstract

**Background:**

Transradial access (TRA) is increasingly preferred over transfemoral access (TFA) for coronary procedures due to its safety profile. However, uptake varies across clinical settings, particularly in complex and high-risk patients.

**Objective:**

To assess arterial access site utilization (TRA vs TFA) and associated clinical and procedural factors in patients undergoing coronary procedures performed by a single operator at a tertiary center.

**Methods:**

We conducted a retrospective review of 250 patients who underwent coronary procedures between September 2024 and February 2025, all performed by a single operator. Demographic, clinical, and procedural data were analyzed to evaluate factors associated with arterial access choice. Logistic regression models were used to determine predictors of access site.

**Results:**

TFA was used in 52.8% and TRA in 47.2% of procedures. TRA was more common in stable patients and for diagnostic angiography (68% vs 32%), while TFA was preferred in emergencies, LV dysfunction, and complex multivessel disease. Independent predictors of TFA included older age (OR 1.021; p = 0.049), mild/moderate LV dysfunction (ORs 2.93 and 3.16, respectively), and prior CABG (p = 0.01). TRA was associated with fewer complications and was never used in unstable patients. Access site choice was also significantly associated with coronary disease burden and treated vessel complexity.

**Conclusion:**

Despite global trends favoring TRA, TFA remains predominant in high-risk and complex cases in our setting. Tailoring access strategies based on patient risk and procedural complexity remains essential.

## Introduction

Over the past two decades, transradial access (TRA) has gained increasing global acceptance for coronary angiography and percutaneous coronary intervention (PCI), even in high-risk and emergent settings, largely due to its superior safety profile and anatomical advantages. The radial artery, being superficial and easily compressible against underlying bone, enables rapid and effective hemostasis, significantly lowering the risk of major bleeding and vascular complications compared to the femoral artery, which lies deeper and is adjacent to critical structures such as the femoral vein and nerves. TRA has been associated with significantly reduced bleeding, improved patient comfort, earlier ambulation, shorter hospital stays, and lower healthcare costs compared to the traditional TFA route [1,2]. Randomized trials and meta-analyses have consistently demonstrated that TRA reduces access site complications, especially in patients with acute coronary syndromes (ACS), and may even improve short-term mortality outcomes in high-risk populations [3–5]

Despite these clear advantages, TFA remains widely used, particularly in certain clinical scenarios. The femoral artery provides a larger and more consistent vascular conduit, capable of accommodating larger sheath sizes and complex catheter systems. The femoral route offers a relatively straight and direct course to the coronary ostia, which can facilitate easier catheter manipulation, particularly in patients with challenging aortic or subclavian anatomy [1]. TFA is often preferred in emergencies or in patients with hemodynamic instability, as it allows for faster vascular access and easier insertion of temporary pacing leads or support devices. Furthermore, unlike the radial artery, the femoral artery is less prone to anatomical variations such as loops or spasm, which can complicate catheter advancement during radial procedures. [2–6]. The choice of access site is also influenced by operator proficiency, institutional culture, and procedural complexity [7].

Although the global trend favors TRA, real-world data from diverse healthcare systems are limited. In many centers, particularly in low-resource or mixed-capability environments, the uptake of TRA may remain modest or selective [8]. This study aims to assess the initial arterial access used for coronary procedures and identify patient- and procedure-related factors associated with access site selection in a real-world setting, based on the experience of a single operator.

## Methods

This retrospective, observational study was conducted at a tertiary Hospital in southern India and included all adult patients who underwent coronary angiography or percutaneous coronary intervention (PCI) between September 1, 2024, and February 28, 2025. All procedures during this period were performed by a single experienced interventional cardiologist following standard institutional protocols.

A total of 250 consecutive patients were included. Patients were eligible if they were aged 18 years or older and had complete medical records related to coronary procedures performed within the study period. Those with incomplete data or who underwent non-coronary interventions, or Patients undergoing procedures by operators other than the designated single operator during the study period, were excluded from the analysis.

Patient data were extracted using a structured collection format, which included demographic characteristics (age, sex), clinical indications (STEMI, NSTEMI, unstable angina, chronic coronary syndrome (CCS)), comorbidities (hypertension, diabetes mellitus, prior PCI or CABG), hemodynamic status at presentation (stable, heart failure, cardiogenic shock), and echocardiographic data such as left ventricular ejection fraction (LVEF). Procedural details recorded included the arterial access site (transradial or transfemoral), procedure type (elective or emergency), treated vessels, use of mechanical circulatory support devices (e.g., intra-aortic balloon pump or Impella), atherectomy device usage, and access site complications observed during hospitalization. All diagnoses, comorbidities, and procedural details (including arterial access site) were verified from hospital medical records, catheterization laboratory reports, and operator notes. No data were obtained solely from patient or caregiver self-report.

Arterial access was categorized based on the primary site used at the start of the procedure. Emergency procedures were defined as unplanned interventions for patients presenting with acute coronary syndromes or hemodynamic compromise. LVEF was classified as normal (>55%), mildly reduced (41–54%), moderately reduced (30–40%), or severely reduced (<30%).

All data were cleaned and entered into Microsoft Excel and analyzed using SPSS version 26.0. Continuous variables were summarized using means and standard deviations, while categorical variables were described using frequencies and percentages. Comparative analyses between transradial and transfemoral access groups were conducted using independent samples t-tests for continuous variables and chi-square tests (or Fisher’s exact test where appropriate) for categorical variables. Bivariate logistic regression was first used to assess potential associations between access site and clinical/procedural variables. Variables with p-values ≤0.05 in univariable analysis, as well as clinically relevant covariates, were entered into a multivariable logistic regression model to identify factors associated with arterial site selection and to adjust for potential confounders. Variables that demonstrated perfect separation (e.g., prior CABG, cardiogenic shock, use of mechanical circulatory support, and atherectomy/calcium-modifying device use) were described separately but excluded from the model. A two-sided p-value <0.05 was considered statistically significant

The study was approved by the institution’s Ethics Review Committee, and as it involved retrospective anonymized data, informed consent was waived. Data was collected from March 18, 2025, to April 15, 2025.

## Results

### Baseline characteristics

A total of 250 patients underwent coronary procedures during the study period, of whom 118 (47.2%) were treated via transradial access (TRA) and 132 (52.8%) via transfemoral access (TFA). The mean age of the study population was 61.65 ± 12.6 years, with patients in the TFA group being significantly older than those in the TRA group (63.30 ± 12.44 vs 59.81 ± 12.65 years; *p* = 0.029). Males comprised 74.0% of the total population, with no significant difference in sex distribution between the two access groups (*p* = 0.290).

Regarding cardiovascular risk factors, there were no statistically significant differences in the prevalence of hypertension (69.5% vs 64.4%; *p* = 0.396), diabetes mellitus (53.4% vs 48.5%; *p* = 0.447), dyslipidemia (23.7% vs 22.0%; *p* = 0.759), between TRA and TFA groups, respectively. Prior PCI was documented in 17.2% of patients, with similar distribution between the access groups (*p* = 0.660). Notably, prior coronary artery bypass grafting (CABG) was significantly more common in the TFA group (6.1%) and absent in the TRA group (*p* = 0.010).

Laboratory values, including mean hemoglobin (13.1 ± 1.5 g/dL vs 12.9 ± 1.7 g/dL; *p* = 0.400) and serum creatinine (1.17 ± 0.49 mg/dL vs 1.25 ± 0.55 mg/dL; *p* = 0.181), did not differ significantly between the groups.

Assessment of left ventricular function revealed that a greater proportion of patients in the TRA group had preserved LVEF (>55%) compared to the TFA group (70.3% vs 45.5%). Conversely, mild (17.8% vs 32.6%; *p* = 0.001) and moderate (7.6% vs 16.7%; *p* = 0.009) LV dysfunction was significantly more frequent in the TFA group. There was no significant difference in the prevalence of severe LV dysfunction (*p* = 0.809). Table 1 depicts Baseline Characteristics, Laboratory Values, and Preprocedural Conditions.

**Table 1:** Baseline characteristics, laboratory values, and preprocedural conditions.

Variable	Total (N = 250)	TRA (n = 118)	TFA (n = 132)	p-value
**Demographics**				
Age, mean ± SD (years)	61.65 ± 12.6	59.81 ± 12.65	63.30 ± 12.44	0.029
Male sex, n (%)	185 (74.0)	91 (77.1)	94 (71.2)	0.290
**Risk Factors**				
Hypertension, n (%)	167 (66.8)	82 (69.5)	85 (64.4)	0.396
Diabetes mellitus, n (%)	127 (50.8)	63 (53.4)	64 (48.5)	0.447
Dyslipidemia, n (%)	57 (22.8)	28 (23.7)	29 (22.0)	0.759
Prior PCI, n (%)	43 (17.2)	19 (16.1)	24 (18.2)	0.660
Prior CABG, n (%)	8 (3.2)	0 (0.0)	8 (6.1)	0.010
Hemoglobin, mean ± SD (g/dL)	13.0 ± 1.6	13.1 ± 1.5	12.9 ± 1.7	0.400
Serum creatinine, mean ± SD (mg/dL)	1.21 ± 0.52	1.17 ± 0.49	1.25 ± 0.55	0.181
**Indications**				
Unstable Angina (UA)	40 (16.0%)	24 (60.0%)	16 (40.0%)	0.045
NSTEMI	62 (24.8%)	23 (37.1%)	39 (62.9%)	0.010
STEMI	60 (24.0%)	23 (38.3%)	37 (61.7%)	0.013
CCS	53 (21.2%)	34 (64.2%)	19 (35.8%)	0.001
Staged PCI	13 (5.2%)	4 (30.8%)	9 (69.2%)	0.153
Others*	22 (8.8%)	10 (45.5%)	12 (54.5%)	0.601
**Procedure Timing and Type**				
Emergency CAG	30 (12.0%)	7 (5.9%)	23 (17.4%)	0.005
Elective PCI	67 (26.8%)	27 (22.9%)	40 (30.3%)	0.168
Emergency PCI	40 (16.0%)	10 (8.5%)	30 (22.7%)	0.002
CAG alone	130 (52.0%)	68 (57.6%)	62 (47.0%)	0.103
CAG with ad hoc PCI	78 (31.2%)	40 (33.9%)	38 (28.8%)	0.411
PCI only (staged)	42 (16.8%)	10 (8.5%)	32 (24.2%)	0.001
**Left Ventricular Function Before the Procedure**				
Normal LVEF (>55%)	143 (57.9%)	83 (70.3%)	60 (45.5%)	
Mild dysfunction (41–54%)	64 (25.9%)	21 (17.8%)	43 (32.6%)	0.001
Moderate dysfunction (30–40%)	31 (12.6%)	9 (7.6%)	22 (16.7%)	0.009
Severe dysfunction (<30%)	9 (3.6%)	5 (4.2%)	4 (3.0%)	0.809
**Preprocedural Patient Condition**				
Stable condition	184 (73.6%)	98 (83.1%)	86 (65.2%)	
Heart failure	52 (20.8%)	20 (16.9%)	32 (24.2%)	0.141
Cardiogenic shock	14 (5.6%)	0 (0.0%)	14 (10.6%)	<0.001

*Includes CAG done as part of the workup for cardiomyopathy, check CAG, Advanced AV block requiring permanent pacemaker

In terms of clinical condition before the procedure, patients undergoing TRA were more likely to be hemodynamically stable at the time of the procedure (83.1% vs 65.2%). Heart failure was more prevalent in the TFA group (24.2% vs 16.9%), though this difference did not reach statistical significance (*p* = 0.141). Cardiogenic shock was observed exclusively in the TFA group (10.6% vs 0.0%; *p* < 0.001).

### Procedural indications, timing, and type

Of the 250 patients, the most common indications were Non–ST-Elevation Myocardial Infarction (NSTEMI) (24.8%), ST-Elevation Myocardial Infarction (STEMI) (24.0%), unstable angina (16.0%), and chronic coronary syndrome (21.2%). Smaller proportions underwent procedures for staged PCI (5.2%) and other indications (8.8%). TRA was more commonly used in CCS and unstable angina cases, while TFA was more frequent in STEMI and NSTEMI. Elective CAG was performed in 45.2% of patients and was more common in the TRA group. Emergency CAG and emergency PCI were more frequently performed via TFA. Elective PCI accounted for 26.8% of procedures and showed no significant access preference alone was performed in 52.0% of cases, ad hoc PCI in 31.2%, and staged PCI in 16.8%. Staged PCI was more common in the TFA group.

#### Baseline characteristics of diagnostic procedures.

The study population consisted of 78 patients undergoing diagnostic coronary angiography (CAG) without percutaneous coronary intervention. Baseline characteristics, categorized by access site (transradial [TRA] n = 53; transfemoral [TFA] n = 25), showed similar demographics between the groups. The average age was 60.1 ± 11.8 years (TRA 58.9 ± 12.1 vs. TFA 62.8 ± 10.9, p = 0.15), with a predominance of males (74.4% overall; TRA 75.5% vs. TFA 72.0%, p = 0.73). Cardiovascular risk factors were similarly distributed, including hypertension (61.5% overall), diabetes mellitus (64.1%), and prior PCI (12.8%). Left ventricular function differed significantly between the groups, with TRA patients more frequently exhibiting normal ejection fraction (>55%; 67.9% vs. 48.0%, p = 0.03). The TRA group had a higher proportion of stable patients (84.9% vs. 68.0%, p = 0.04), while TFA was more commonly used for patients with heart failure (24.0% vs. 11.3%) or cardiogenic shock (8.0% vs. 3.8%), though these differences were not statistically significant. Angiographic findings indicated that TRA was associated with more frequent normal coronary arteries (28.3% vs. 12.0%, p = 0.04). A trend toward a greater prevalence of triple-vessel disease in the TFA group (24.0% vs. 11.3%, p = 0.18) did not reach statistical significance. Procedure timing (elective vs. emergency) did not differ between the groups. Complications were infrequent overall. Radial artery spasm occurred in 3.8% of TRA cases, while one pseudoaneurysm (1.3%) was noted in the TFA group. One mortality (4.0%) was recorded in the TFA group compared to none in the TRA group (p = 0.15), see table 2.

**Table 2:** Baseline characteristics of patients undergoing diagnostic coronary procedures.

Variable	Overall (N = 78)	TRA (N = 53)	TFA (N = 25)	p-value
**Demographics**				
Age (years), mean ± SD	60.1 ± 11.8	58.9 ± 12.1	62.8 ± 10.9	0.15
Male sex, n (%)	58 (74.4%)	40 (75.5%)	18 (72.0%)	0.73
**Risk Factors**				
Hypertension (any), n (%)	48 (61.5%)	32 (60.4%)	16 (64.0%)	0.75
Diabetes Mellitus (any), n (%)	50 (64.1%)	34 (64.2%)	16 (64.0%)	0.99
Prior PCI, n (%)	10 (12.8%)	6 (11.3%)	4 (16.0%)	0.55
Prior CABG, n (%)	0 (0%)	0 (0%)	0 (0%)	–
**Left Ventricular Function**				
Normal (>55%), n (%)	48 (61.5%)	36 (67.9%)	12 (48.0%)	**0.03**
Mild Dysfunction (41–54%), n (%)	20 (25.6%)	11 (20.8%)	9 (36.0%)	
Moderate/Severe Dysfunction (≤40%), n (%)	10 (12.8%)	6 (11.3%)	4 (16.0%)	
**Procedure Timing**				
Elective CAG, n (%)	47 (60.3%)	32 (60.4%)	15 (60.0%)	0.97
Emergency CAG, n (%)	11 (14.1%)	6 (11.3%)	5 (20.0%)	0.31
**Patient Condition**				
Stable, n (%)	62 (79.5%)	45 (84.9%)	17 (68.0%)	**0.04**
Heart Failure (Class II-III), n (%)	12 (15.4%)	6 (11.3%)	6 (24.0%)	
Cardiogenic Shock, n (%)	4 (5.1%)	2 (3.8%)	2 (8.0%)	
**CAG Findings**				
Normal Coronaries, n (%)	18 (23.1%)	15 (28.3%)	3 (12.0%)	**0.04**
Single-Vessel Disease (SVD), n (%)	22 (28.2%)	15 (28.3%)	7 (28.0%)	0.98
Triple-Vessel Disease (TVD), n (%)	12 (15.4%)	6 (11.3%)	6 (24.0%)	0.18
Left Main (LM) Disease, n (%)	8 (10.3%)	5 (9.4%)	3 (12.0%)	0.71
**Complications**				
Radial Artery Spasm, n (%)	2 (2.6%)	2 (3.8%)	0 (0%)	0.32
Hematoma/Pseudoaneurysm, n (%)	1 (1.3%)	0	1 (4.0%)	0.15
Mortality	1 (1.3%)	0	1 (4.0%)	0.15

### Comparison of TRA vs TFA in interventional procedures

A total of 172 patients underwent interventional coronary procedures, with 65 (37.8%) receiving transradial access (TRA) and 107 (62.2%) receiving transfemoral access (TFA). The mean age was significantly higher in the TFA group (63.6 ± 12.6 years) compared to the TRA group (60.2 ± 13.1 years, p = 0.04). The proportion of male patients was comparable between the groups (TRA 78.5% vs. TFA 71.0%, p = 0.28). Comorbidities such as hypertension, diabetes, dyslipidemia, and prior PCI were similarly distributed between groups (p > 0.05). However, prior CABG was significantly more frequent in the TFA group (7.5%) compared to none in the TRA group (p = 0.02). Left ventricular function significantly influenced access site selection. Normal LVEF (>55%) was present in 89.2% of TRA cases vs. 37.4% in TFA, while mild and moderate dysfunction were more common in the TFA group (p < 0.001). Regarding preprocedural clinical status, TRA was predominantly used in stable patients (89.2% vs. 63.6%, p = 0.001), while heart failure and shock were more common in the TFA group. Notably, all 10 patients with shock underwent procedures via TFA. Elective procedures were more frequently performed via TRA (63.1% vs. 47.7%, p = 0.045), while TFA predominated in emergency interventions. In terms of clinical indications, unstable angina was significantly more often treated via TRA (27.7% vs. 13.1%, p = 0.02). Other indications, such as NSTEMI, STEMI, and CCS, were similarly distributed. Staged PCI was more frequent in the TFA group (17.8% vs. 4.6%, p = 0.01). Anatomic and procedural complexity favored femoral access. Double-vessel disease was more common in TFA (24.3% vs. 10.8%, p = 0.02), while single-vessel disease was more frequent in TRA (78.5% vs. 62.6%, p = 0.03). There were no significant differences in the target coronary territories treated (LAD, RCA, LCX), though graft interventions occurred only in the TFA group. TRA was associated with significantly fewer lesions treated per patient (mean 1.22 vs. 1.41, p = 0.026). All advanced techniques (rotational/orbital atherectomy, Intravascular Lithotripsy (IVL)) and mechanical circulatory support (MCS), including intra-aortic balloon pump and Impella, were performed via TFA only, with MCS usage significantly higher in TFA (p = 0.021). Access-site complications were rare overall. All cases of hematoma and pseudoaneurysm occurred in the TFA group. One case of radial artery spasm (1.5%) was noted in the TRA group. Finally, all in-hospital deaths (n = 5, 2.9%) occurred in the TFA group, with no mortality in the TRA group (p = 0.04), see Table 3.

**Table 3:** Comparison of interventional procedures by arterial access site (TRA vs. TFA).

Variable	Total (N = 172)	TRA (n = 65)	TFA (n = 107)	p-value
**Demographics and Risk Factors**				
Age (years, mean ± SD)	62.3 ± 12.9	60.2 ± 13.1	63.6 ± 12.6	0.04
Male sex (%)	127 (73.8%)	51 (78.5%)	76 (71.0%)	0.28
Hypertension (%)	107 (62.2%)	40 (61.5%)	67 (62.6%)	0.89
Diabetes (%)	106 (61.6%)	37 (56.9%)	69 (64.5%)	0.32
Dyslipidemia (%)	87 (50.6%)	32 (49.2%)	55 (51.4%)	0.79
Prior PCI (%)	33 (19.2%)	13 (20.0%)	20 (18.7%)	0.84
Prior CABG (%)	8 (4.7%)	0 (0%)	8 (7.5%)	0.02
Left Ventricular Function				
Normal LVEF (>55%)	98 (57.0%)	58 (89.2%)	40 (37.4%)	<0.001
Mild dysfunction (41–54%)	44 (25.6%)	5 (7.7%)	39 (36.4%)	
Moderate dysfunction (30–40%)	22 (12.8%)	2 (3.1%)	20 (18.7%)	
**Preprocedural Condition**				
Stable	126 (73.3%)	58 (89.2%)	68 (63.6%)	0.001
Heart Failure (Class II–III)	36 (20.9%)	7 (10.8%)	29 (27.1%)	
Shock	10 (5.8%)	0 (0%)	10 (9.3%)	
**Procedure Urgency**				
Elective	92 (53.5%)	41 (63.1%)	51 (47.7%)	0.045
Emergency	80 (46.5%)	24 (36.9%)	56 (52.3%)	
**Clinical Indications**				
Unstable Angina (UA)	32 (18.6%)	18 (27.7%)	14 (13.1%)	0.02
NSTEMI	36 (20.9%)	13 (20.0%)	23 (21.5%)	0.82
STEMI	60 (34.8%)	23(35.3%)	37 (34.5%)	0.89
Chronic Coronary Syndrome (CCS)	52 (30.2%)	20 (30.8%)	32 (29.9%)	0.91
Staged PCI	22 (12.8%)	3 (4.6%)	19 (17.8%)	0.01
**Coronary Disease Pattern and Treated Territories**				
Left Main Disease (%)	14 (8.1%)	4 (6.2%)	10 (9.3%)	0.45
Single-Vessel Disease	118 (68.6%)	51 (78.5%)	67 (62.6%)	0.03
Double-Vessel Disease	33 (19.2%)	7 (10.8%)	26 (24.3%)	0.02
Triple-Vessel Disease	5 (2.9%)	1 (1.5%)	4 (3.7%)	0.40
**Choice of Arterial Access in Relation to Target Vessel**				
LAD Intervention (%)	94 (54.7%)	35 (53.8%)	59 (55.1%)	0.87
RCA Intervention (%)	57 (33.1%)	17 (26.2%)	40 (37.4%)	0.13
LCX Intervention (%)	44 (25.6%)	15 (23.1%)	29 (27.1%)	0.55
Graft Intervention (%)	2 (1.2%)	0 (0%)	2 (1.9%)	0.20
Lesions per patient (mean)	1.32	1.22	1.41	0.026
**Advanced Techniques Used**				
Rotational Atherectomy (%)	5(3%)	0 (0%)	5(4.5%)	0.08
Orbital Atherectomy (%)	3 (1.8%)	0 (0%)	3(2.7%)	0.35
Intravascular Lithotripsy (%)	3(1.8%)	0 (0%)	(2.7%)	0.35
Any MCS	5 (2.9%)	0 (0%)	5 (4.7%)	0.021
IABP	4 (2.3%)	0 (0%)	4 (3.7%)	—
Impella	1 (0.6%)	0 (0%)	1 (0.9%)	—
**Access-Site Complications**				
Minor Hematoma (%)	1 (0.6%)	0 (0%)	1 (0.9%)	0.35
Major Hematoma (%)	1 (0.6%)	0 (0%)	1 (0.9%)	0.35
Pseudoaneurysm (%)	2 (1.2%)	0 (0%)	2 (1.9%)	0.20
Radial Artery Spasm (%)	1 (0.6)	1 (1.5%)	0 (0%)	0.20
**In-Hospital Outcomes**				
In-hospital Mortality (%)	5 (2.9%)	0	5 (4.7%)	0.04

On multivariable logistic regression (Table 4.), older age, mild and moderate LV dysfunction, and emergency procedures were independently associated with increased likelihood of transfemoral access. Prior CABG, cardiogenic shock, use of mechanical circulatory support, and atherectomy/calcium-modifying devices were seen exclusively in the transfemoral group and therefore could not be analyzed in the regression model. STEMI diagnosis showed a borderline association with transfemoral access, while severe LV dysfunction, heart failure at presentation, and number of lesions treated were not significant after adjustment.

**Table 4:** Multivariable logistic regression analysis of predictors of arterial access site selection.

Predictor	Adjusted OR	95% CI	p-value
Age (per year increase)	1.02	1.00–1.04	0.049
History of CABG*	–	–	–
**Indication**			
– Unstable angina	1.20	0.60–2.40	0.580
– NSTEMI	1.85	0.95–3.58	0.070
– STEMI	1.72	0.94–3.16	0.080
**Procedure type**			
– Emergency CAG/PCI	2.15	1.25–3.71	0.006
– Staged PCI	1.32	0.55–3.17	0.530
**Left ventricular ejection fraction**			
– Mild dysfunction	2.93	1.56–5.50	0.001
– Moderate dysfunction	3.16	1.33–7.52	0.009
– Severe dysfunction	0.85	0.22–3.31	0.809
**Preprocedural condition**			
Heart failure	1.58	0.86–2.91	0.141
Cardiogenic shock*	–	–	–
Use of MCS*	–	–	–
Advanced techniques used*	–	–	–

*Variables not included in the final multivariable model due to collinearity or low event frequency. All cases used TFA.

## Discussion

Coronary artery disease (CAD) remains a leading cause of global morbidity and mortality, accounting for ≈9 million deaths annually [9].Percutaneous coronary intervention (PCI) has evolved into a cornerstone of CAD management since its inception in 1977, fundamentally transforming outcomes for patients with ACS and complex coronary anatomy. [10].

The selection of arterial access for coronary procedures is a critical determinant of procedural outcomes, patient safety, and overall clinical efficiency. Traditionally, the femoral artery was the predominant access site; however, over the past two decades, the radial artery has gained widespread adoption due to its association with significantly reduced rates of access-site bleeding, vascular complications, and mortality, particularly in high-risk populations. Clinical trials have demonstrated that radial access not only improves short-term outcomes but also enhances patient comfort and facilitates early mobilization, leading to shorter hospital stays and improved patient satisfaction [5].

In the present study, transradial access (TRA) was utilized in 47.3% of all coronary procedures. This is consistent with findings from a seven-year retrospective study conducted in North India, which reported a TRA utilization rate of 44.35% over the study period [8] However, it is important to note that the latter study was conducted over a decade ago, and the adoption of TRA has likely increased since then. Over the past two decades, TRA has progressively replaced TFA as the preferred route for both diagnostic CAG and PCI, owing to robust evidence demonstrating its superior safety profile. Recent registry data suggest that TRA is employed in 50–80% of coronary procedures in high-volume centers across Europe, North America, and Asia. Furthermore, current national (Indian) and international clinical guidelines advocate for a radial-first approach in both acute coronary syndrome (ACS) and chronic coronary syndrome (CCS) settings due to its association with reduced vascular complications and improved patient outcomes [7,11–13].

Analysis of the available data shows that the TRA had a success rate of 96.6% for both diagnostic procedures and PCIs, the majority of which were ad hoc PCIs. This rate is slightly lower than the 99.07% TRA success rate reported in a study from Northern India a decade ago, but remains comparable to findings from major international trials. For instance, the RIVAL trial and MATRIX trial reported TRA crossover rates of 7% and 7.9%, respectively, reflecting the procedural challenges occasionally associated with radial access [5,8,14].

The mean age of patients in our study was higher (61.65 ± 12.6 years) compared to that reported in the earlier North Indian study (56.80 ± 9.86 years) [8] Notably, patients who underwent coronary procedures via the transfemoral route were significantly older than those treated through the transradial approach. Given that advanced age is a known risk factor for vascular and bleeding complications, this demographic trend underscores the importance of access site selection. In the RIVAL trial, a subgroup analysis of patients aged 75 years or older demonstrated a favorable trend toward improved outcomes with transradial access compared to transfemoral access. Among elderly patients, the composite primary outcome of death, myocardial infarction, stroke, or non-CABG-related major bleeding occurred in 8.1% of patients in the TRA group versus 9.1% in the TFA group (HR 0.89; 95% CI 0.58–1.34), indicating a potential clinical benefit of TRA in this high-risk subgroup, although the interaction was not statistically significant (p for interaction = 0.57). [5].In our study, the majority of patients with a history of prior PCI underwent procedures via the TFA, although this difference was not statistically significant. Similarly, all patients with a history of CABG) were managed through TFA. This is comparable with findings from a large Indian registry, in which 824 patients (3.4%) had prior CABG; the majority of these patients underwent procedures via TFA, while only 13 were treated through the TRA [8]. While our study reflects a predominant use of TFA in patients with prior revascularization, current evidence from randomized controlled trials and international guidelines supports the preferential use of TRA, particularly in patients with prior PCI. The **RIVAL trial** demonstrated that TRA was associated with significantly lower rates of complications without compromising procedural success, even in complex cases such as prior PCI. The **MATRIX trial** further reinforced these findings by linking TRA to lower all-cause mortality and bleeding in ACS patients undergoing invasive management [5,14].

For patients with prior CABG, TRA—particularly via the left radial artery—has gained favor for selective LIMA graft engagement, as supported by observational studies and expert consensus. However, challenges such as altered anatomy, graft orientation, and procedural complexity may lead operators to prefer TFA in routine practice. In our cohort, the exclusive use of TFA in CABG patients likely reflects procedural familiarity, graft anatomy considerations, and institutional protocol. In our study, patients with preserved LVEF were more frequently treated via the TRA), while those with mild to moderate LV dysfunction underwent procedures predominantly through the TFA). Similar patterns have been observed in larger registries, where reduced LVEF was an independent predictor of femoral access selection, likely reflecting operator caution in hemodynamically compromised patients [4,15]. However, evidence from observational studies supports the safety and efficacy of TRA even in patients with significant LV dysfunction. Mamas et al. demonstrated that TRA in cardiogenic shock was associated with lower 30-day mortality and bleeding, particularly in experienced centers [4,15]. Despite the technical challenges, TRA remains a viable option in selected patients with reduced systolic function.

In our study, TFA was more frequently used in emergency and hemodynamically unstable patients, which is consistent with previous studies. Mamas et al. reported a similarly high use of TFA (74%) in patients with cardiogenic shock in the UK, often due to operator familiarity and the need for rapid or large-bore access [4,16]. The landmark nationwide Dutch study by Peters et al., encompassing 1,562 cardiogenic shock patients undergoing PCI, revealed that while transfemoral access (TFA) remained prevalent in cases with extreme hemodynamic compromise (evidenced by lower mean arterial pressure, elevated lactate levels, and frequent mechanical circulatory support requirements), TRA was associated with dramatically superior outcomes. [6]. These findings challenge traditional assumptions about access-site selection in critically ill patients, suggesting that the radial approach may offer life-saving benefits beyond simply reducing vascular complications. This real-world evidence complements randomized trial data and supports guideline recommendations favoring TRA as the default strategy, while acknowledging that individualized decision-making remains essential for patients requiring large-bore access for hemodynamic support devices. The SAFARI-STEMI trial also showed comparable outcomes between access routes in STEMI patients but noted more frequent crossover from TRA to TFA in acute cases [17].

Although our study found that patients with complex coronary artery disease, such as double-vessel, triple-vessel, and left main involvement, were more frequently treated via the transfemoral approach (TFA), which is consistent with previous studies showing a preference for femoral access in anatomically or procedurally complex cases, there is growing evidence that the transradial approach (TRA) can be safely and effectively used in these same high-complexity scenarios. This shift is supported by recent meta-analyses and large trials such as RIVAL and MATRIX, which demonstrate that with increasing operator experience and advancements in guiding equipment, TRA is not only feasible but also associated with reduced vascular complications, even in patients undergoing high-risk or multivessel interventions [3,8].

In our study, all atherectomy procedures (n = 11) and all cases requiring mechanical circulatory support — including IABP (n = 4) and Impella (n = 1) — were performed via the transfemoral approach (TFA). This is consistent with standard clinical practice, given the larger sheath requirements and superior guide catheter support provided by femoral access, which are often necessary for plaque modification devices and high-risk percutaneous coronary interventions (PCI).

However, growing evidence demonstrates that transradial access (TRA) is a feasible and safe alternative for selected cases of rotational atherectomy, especially with smaller burr sizes (≤1.75 mm) and the use of 6 or 7 Fr guiding catheters. A large systematic review and meta-analysis by Khan et al. (2018), which included over 9,000 patients, found that TRA was associated with comparable procedural success, mortality, and major adverse cardiac events (MACE) to TFA, while also offering significantly reduced bleeding and vascular complications.

Similarly, Ferstl et al. (2022) reported in their cohort of 427 patients that TRA achieved a 97% procedural success rate for rotational atherectomy, with no significant difference in burr size or outcome compared to TFA. Notably, access site complications and major bleeding events were significantly lower in the TRA group (4%) compared to TFA (13%). While mechanical circulatory support and larger burrs still necessitate femoral access in most centers, their findings highlight the growing role of TRA for complex coronary interventions, particularly in high-volume radial centers [18,19]

In our study, when radial artery spasm is included as an access-site–related complication, the overall complication rate in the TRA group was 2.54% (3/118. Importantly, no hematomas or pseudoaneurysms were observed in the TRA group. By contrast, the transfemoral access (TFA) group exhibited a higher overall complication rate of 3.03% (4/132), including minor hematoma (0.76%), major hematoma (0.76%), and pseudoaneurysm (1.52%). Of the two pseudoaneurysm cases, one was successfully managed with ultrasound-guided compression, while the other resulted in-patient death during the compression attempt. These findings are broadly consistent with those reported by Tewari et al.Notably, the serious femoral complication rate in our study (3.03%) was also higher than that reported by Tewari et al., where major hematomas and pseudoaneurysms occurred in 0.2% and 0.14% of femoral cases, respectively.[8]

Further corroborating our findings, the meta-analysis by Chiarito et al. (2021) demonstrated that TRA significantly reduces major bleeding (OR 0.53), vascular complications (OR 0.32), and all-cause mortality (OR 0.74) compared to TFA, with consistent benefit across diverse patient populations [3]. Similarly, in the SAFARI-STEMI trial, although overall access-site complications were low, the femoral group showed a higher incidence of retroperitoneal bleeding, and bleeding complications were more common in the absence of vascular closure devices [17]. Collectively, these findings reinforce the safety advantage of TRA, particularly when performed by experienced operators. Although TRA is not devoid of technical challenges, such as radial spasm, these were non-fatal and manageable in our cohort.

Major bleeding complications are a significant cause of mortality in patients with cardiogenic shock. The presence of cardiogenic shock is associated with a 4-fold increase in major bleeding rates compared to those without shock.

This elevated bleeding risk arises from several factors. Beyond mechanical circulatory support, abnormalities in platelet physiology and coagulation/fibrinolysis pathways following cardiogenic shock may further contribute to bleeding risk [4].

Major bleeding complications are a powerful predictor of mortality, with a substantial proportion occurring at the vascular access site. The use of TRA in PCI procedures has been linked to reductions in both access-site bleeding and mortality. In contrast, among patients with MI and cardiogenic shock treated with an IABP, bifemoral access has been shown to nearly double the risk of major bleeding compared to a single femoral puncture. Therefore, minimizing groin punctures, such as through TRA, may be an important strategy to mitigate bleeding complications and improve outcomes in this high-risk population [4,20,21].

## Conclusion

This study reinforces the growing body of evidence supporting transradial access (TRA) as the preferred approach for coronary procedures, demonstrating its safety, efficacy, and clinical advantages over transfemoral access (TFA) across diverse patient populations. Our findings align with global registry data and randomized trials, showing that TRA is associated with lower complication rates, reduced bleeding, and improved outcomes, even in high-risk subgroups such as elderly patients, those with prior PCI, and individuals with reduced left ventricular function.

Despite these benefits, TFA remains prevalent in complex cases, including those requiring mechanical circulatory support, large-bore access, or prior CABG, reflecting operator familiarity, anatomical challenges, and institutional protocols. However, emerging evidence suggests that with increasing operator expertise and technological advancements, TRA can be successfully employed even in high-complexity interventions, further minimizing vascular risks.

### Strengths and limitations of the study

This study has several notable strengths. Conducted in a high-volume tertiary center, it offers a contemporary, real-world perspective on arterial access practices in both diagnostic and interventional coronary procedures. The inclusion of high-risk subgroups—such as patients with cardiogenic shock, prior CABG, and complex coronary anatomy—enhances the clinical relevance of the findings. By comparing transradial (TRA) and transfemoral access (TFA) in terms of procedural success, complications, and in-hospital outcomes, the study provides meaningful insights into access-site strategy.

However, certain limitations should be acknowledged. The retrospective, cross-sectional, single-center design limits generalizability and precludes establishing causal relationships between patient or procedural characteristics and access-site choice. Access-site selection was operator-dependent rather than randomized, introducing potential selection bias. Crossover between access sites was not captured, possibly underestimating technical challenges with TRA. All patients with cardiogenic shock and prior CABG were treated via TFA, precluding outcome comparison in these groups. Additionally, the study focused only on in-hospital outcomes, without long-term follow-up. although consecutive patients were included, the sample size was modest. Third, several potentially relevant procedural variables—including type of P2Y12 inhibitor, use of glycoprotein IIb/IIIa inhibitors, sheath size and length, detailed lesion characteristics (such as bifurcation or calcification), and duration of hospital stay—were not consistently documented in retrospective records Although patients with complex coronary anatomy such as left main disease and double- or triple-vessel disease were included in this study, detailed lesion-level characteristics (e.g., bifurcation involvement, chronic total occlusions, and lesion length) were not consistently documented and therefore could not be analyzed.

## Supporting information

S1 File Supporting data Plos dataset S1. (XLSX)

S2 File Codebook. (XLSX)
